# Induction immunochemotherapy followed by definitive chemoradiotherapy and consolidation immunotherapy for unresectable stage III non-small cell lung cancer: a multi-institutional retrospective cohort study

**DOI:** 10.3389/fimmu.2025.1602082

**Published:** 2025-07-31

**Authors:** Zhixue Fu, Xin Dong, Jiawen Sun, Wei Deng, Yuting Zhao, Dan Yang, Leilei Jiang, Xiao Chang, Rong Yu, Anhui Shi, Huiming Yu, Jidong Wang, Wei Jiang, Jiawei Lu, Dongjie Chen, Jun Liang, Weihu Wang

**Affiliations:** ^1^ Key Laboratory of Carcinogenesis and Translational Research (Ministry of Education/Beijing), Department of Radiation Oncology, Peking University Cancer Hospital & Institute, Beijing, China; ^2^ Department of Radiation Oncology, Peking University International Hospital, Beijing, China; ^3^ National Cancer Center/National Clinical Research Center for Cancer/Cancer Hospital & Shenzhen Hospital, Chinese Academy of Medical Sciences and Peking Union Medical College, Shenzhen, China

**Keywords:** non-small cell lung cancer, induction immunochemotherapy, consolidation immunotherapy, chemoradiotherapy, real world

## Abstract

**Background:**

For unresectable stage III non-small cell lung cancer (NSCLC), the standard regimen is definitive concurrent chemoradiotherapy (CRT) followed by consolidation immunotherapy. We investigated whether incorporating induction immunochemotherapy enhances the efficacy.

**Materials and methods:**

From June 2018 to December 2022, 294 patients with unresectable stage III NSCLC were included, who did (162, I-CRT-I group) or did not (132, CRT-I group) receive induction immunochemotherapy, followed by definitive CRT and consolidation immunotherapy. Propensity score matching (PSM) adjusted for potential confounding variables. Overall survival (OS), progression-free survival (PFS), recurrence pattern, and safety were evaluated.

**Results:**

After PSM, 206 patients (103 in each group) were included. The median follow-up time was 32.3 and 44.6 months for the I-CRT-I and CRT-I group, respectively. The I-CRT-I group showed a significant improvement in OS compared with the CRT-I group (*p*=0.004). The median OS in the I-CRT-I group was not reached, with 1-, 2-, and 3-year OS rates of 91.3%, 80.0%, and 72.9%, respectively; the CRT-I group had a median OS of 39.3 months, with survival rates of 91.1%, 69.3%, and 52.0%, respectively. PFS (*p*=0.332) and local locoregional recurrence (*p*=0.940) were not significantly different between the groups, while significantly lower cumulative distant metastasis (DM) was noted for the I-CRT-I group (*p*=0.004). Prior to PSM, the adverse events rate was 96.3% and 95.5% in the I-CRT-I and CRT-I groups, respectively, with pneumonitis noted in 57.4% and 58.3%, respectively.

**Conclusion:**

Induction immunochemotherapy followed by definitive CRT and consolidation immunotherapy may improve OS and decrease DM, along with manageable safety.

## 1 Introduction

Non-small cell lung cancer (NSCLC) constitutes approximately 80%–85% of lung cancer cases. Moreover, around one-third of patients with NSCLC are diagnosed at stage III, indicating advanced disease (1). According to the PACIFIC study, the standard of care for unresectable stage III NSCLC is definitive chemoradiotherapy (CRT) followed by consolidation immunotherapy with durvalumab (2–6). Although the PACIFIC study reported long-term benefits from consolidation immunotherapy, with a 5-year overall survival (OS) rate of 42.9% and a progression-free survival (PFS) rate of 33.1%, approximately 57% of patients still succumbed to the disease, and 67% experienced disease progression (6). Therefore, it is essential to explore novel treatment measures that could further enhance the prognoses of patients with unresectable stage III NSCLC. Recently, compelling evidence has emerged, demonstrating the potential advantages of neoadjuvant immunotherapy (7–11). The Checkmate 816 trial showed that neoadjuvant immunochemotherapy significantly extended event-free survival (median: 31.6 months vs. 20.8 months; *p* = 0.005) and increased the proportion of patients achieving a pathological complete response (PCR: 24% vs. 2.2%; *p* < 0.005) among those with resectable NSCLC (7). The primary advantage of induction immunotherapy is that it engages a healthy immune system, unaffected by chemotherapy or radiotherapy (12, 13). A preclinical study also showed that strong activation of the immune system could yield more effective immune surveillance against micrometastatic diseases (14). The clinical phase II AFT-16 trial (15) and KEYNOTE-799 trial (16) revealed that induction immunotherapy prior to definitive CRT was well tolerated and might improve survival in patients with stage III NSCLC. Thus, the approach of induction immunochemotherapy followed by definitive CRT merits further investigation.

Therefore, this study was designed to analyze the efficacy and safety of induction immunochemotherapy followed by definitive CRT and consolidation immunotherapy in patients with unresectable stage III NSCLC. To account for potential confounding factors and ensure the reliability of our findings, we adopted the propensity score matching (PSM) method (17).

## 2 Materials and methods

### 2.1 Patient population

The patents with stage III NSCLC, who received definitive CRT and consolidation immunotherapy with or without induction immunochemotherapy from June 2018 to December 2022 in Peking University Cancer Hospital, Peking University International Hospital, and Cancer Hospital & Shenzhen Hospital Chinese Academy of Medical Sciences, were retrospectively reviewed. The inclusion criteria were as follows: (1) age ≥ 18 years; (2) histologically or cytologically confirmed NSCLC; (3) stage III NSCLC according to the 8th American Joint Committee on Cancer (AJCC) staging system; (4) receiving definitive CRT and consolidation immunotherapy (at least one cycle) with or without induction immunotherapy (at least one cycle). The exclusion criteria were as follows: (1) receiving active or previous autoimmune disease (within the past 2 years) or a history of primary immunodeficiency; (2) receiving radical resection of lung cancer; (3) epidermal growth factor receptor (EGFR) sensitive mutations, anaplastic lymphoma kinase (ALK) gene fusion, and c-ros oncogene 1 (ROS1) gene fusion. This is a multi-institutional retrospective cohort study, so patient demographic and clinical characteristics were extracted from medical history, such as age, sex, Eastern Cooperative Oncology Group (ECOG) status, smoking history, histology, staging, PD-L1 expression and immune checkpoint inhibitors (ICIs).

### 2.2 Treatment

Patients with unresectable stage III NSCLC were categorized into two groups: induction immunochemotherapy followed by definitive CRT and consolidation immunotherapy (I-CRT-I group) and definitive CRT followed by consolidation immunotherapy (CRT-I group). The ICIs included the programmed death ligand-1 (PD-L1) inhibitor and the programmed death-1 (PD-1) inhibitor. The induction, concurrent, and consolidation chemotherapy regimens were platinum-based. All patients received radiotherapy dose of 60-66Gy in 25–33 fractions, and radiotherapy techniques included intensity-modulated radiation therapy (IMRT) or volumetric-modulated arc therapy (VMAT). Radiation therapy simulation was conducted utilizing 4-dimensional computed tomography (4D-CT) scans for all enrolled patients. The gross target volume (GTV) of the primary tumor (GTVp) was delineated based on the primary tumor identified on simulation CT images, while the GTV of the lymph nodes (GTVnd) was defined as any regionally involved lymph nodes exhibiting a short axis greater than 1 cm on pretreatment CT scans or demonstrating high fluorodeoxyglucose uptake on positron emission tomography/computed tomography (PET/CT) scans. For patients who underwent induction immunotherapy or induction chemotherapy, the GTVp and GTVnd were adjusted to include the post-induction tumor volume as observed on 4D-CT. The clinical target volume (CTV) was established by incorporating a 0.5 cm margin beyond the combined GTV (GTVp plus GTVnd) and the pretreatment involved hilar and mediastinal nodal regions, even in cases where enlarged lymph nodes regressed following induction therapy. The planning target volume (PTV) was further expanded by adding a 0.5 cm margin beyond the CTV. Patients were followed up every 3 months during the first 2 years and then every 6 months for the next years. The last follow-up was on November 10, 2024.

### 2.3 End points and assessments

In both groups, the primary endpoints were OS and PFS. We also assessed the objective response rate (ORR) and disease control rate (DCR) of induction ICI combined with chemotherapy. OS was defined as the time elapsed between the last radiotherapy session and death from any cause. PFS was defined as the time elapsed between the last radiotherapy session and the first documented event of tumor progression or death from any cause. PFS was assessed according to the Response Evaluation Criteria in Solid Tumors (RECIST) (version1.1). Based on RECIST (version1.1), ORR was defined as partial response (PR) plus complete response (CR), while DCR was defined as PR and CR plus stable disease (SD). Secondary endpoint was recurrence pattern and safety. Locoregional recurrence (LRR) was defined as the site of recurrence in the primary tumor or the ipsilateral hilum, mediastinum, or supraclavicular area. Distant metastasis (DM) was defined as the site of recurrence in distant sites or non-regional lymph nodes. The toxicities were graded using the Common Terminology Criteria for Adverse Events Version (CTCAE) (version5.0).

### 2.4 Statistical analysis

The two-group baseline patient and tumor characteristics were compared by the chi-square test. PSM was employed to adjust for any imbalanced confounders. The propensity score for each patient was estimated with a logit model that included the following variables: sex, age, ECOG performance status (PS), smoking history, histology, stage, and radiation dose. Two comparable groups were created with 103 patients in each, using a caliper setting of 0.05 and 1:1 allocation. OS and PFS were analyzed by the Kaplan-Meier method and compared by the log-rank test. Median follow-up was estimated with the use of the reverse Kaplan-Meier method. To properly evaluate the patterns of failure, the site of recurrence (locoregional or distant) was analyzed by considering death as a competing risk, respectively. Univariate Cox regression model was used to calculate hazard ratio (HR) and 95% confidence interval (CI). All tests were two sided, and statistical significance was set at a p value <0.05. Statistical analyses were performed using SPSS (version 27.0) and R software (version 4.2.2).

## 3 Results

### 3.1 Baseline patient characteristics

**Figure 1:**
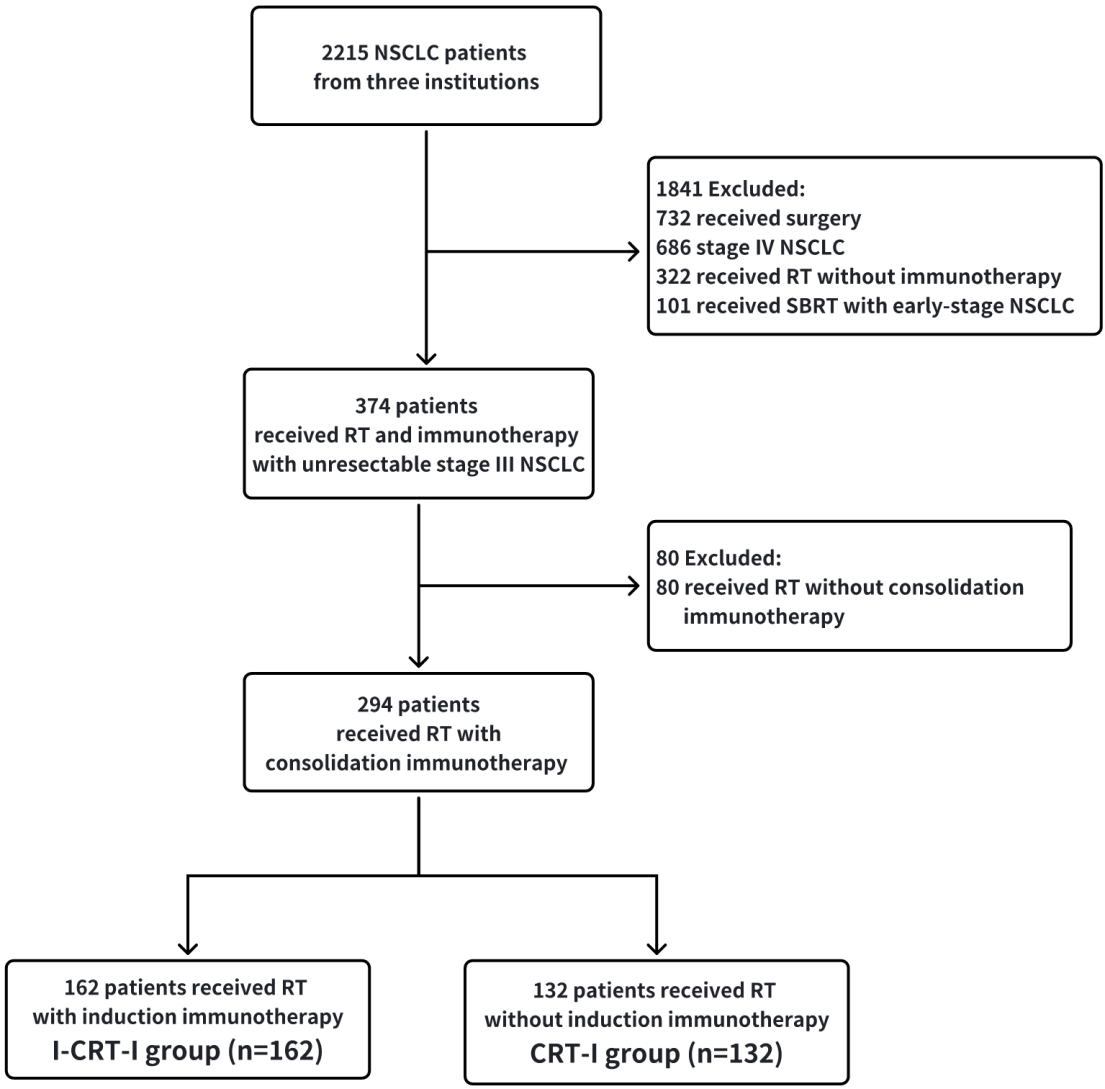
Patient inclusion flow chart.

This study enrolled 294 patients, including the I-CRT-I (n=162) and CRT-I groups (n=132) (**Figure 1**). After PSM adjustment, 206 patients were analyzed, including 103 in the I-CRT-I group and 103 in the CRT-I group. The demographic and therapeutic characteristics before and after PSM adjustment are presented in **Table 1**, with further details in **Table 2**.

**Table 1:** Patients' characteristics and treatment information before and after PSM.

Characteristics	Before PSM				After PSM			
	Overall	I-CRT-I group	CRT-I group	*P*	Overall	I-CRT-I group	CRT-I group	*P*
	N=294, (%)	N=162, (%)	N=132, (%)		N=206, (%)	N=103, (%)	N=103, (%)	
Sex				0.171				0.421
Male	262 (89.1%)	148 (91.4%)	114 (86.4%)		191 (92.7%)	94 (91.3%)	97 (94.2%)	
Female	32 (10.9%)	14 (8.6%)	18 (13.6%)		15 (7.3%)	9 (8.7%)	6 (5.8%)	
Age (years)				0.131				0.567
≤65	181 (61.6%)	106 (65.4%)	75 (56.8%)		126 (61.2%)	61 (59.2%)	65 (63.1%)	
>65	113 (38.4%)	56 (34.6%)	57 (43.2%)		80 (38.8%)	42 (40.8%)	38 (36.9%)	
ECOG PS				0.810				>0.999
0	192 (65.3%)	103 (63.6%)	89 (67.4%)		146 (70.9%)	73 (70.9%)	73 (70.9%)	
1	99 (33.7%)	57 (35.2%)	42 (31.8%)		60 (29.1%)	30 (29.1%)	30 (29.1%)	
2	3 (1.0%)	2 (1.2%)	1 (0.8%)			0	0	
Smoking				0.280				0.836
Yes	248 (84.4%)	140 (86.4%)	108 (81.8%)		179 (86.9%)	90 (87.4%)	89 (86.4%)	
No	46 (15.6%)	22 (13.6%)	24 (18.2%)		27 (13.1%)	13 (12.6%)	14 (13.6%)	
Histology				0.004				0.784
Squanous	202 (68.7%)	121 (74.7%)	81 (61.4%)		151 (73.3%)	77 (74.8%)	74 (71.8%)	
Adenocarcinoma	78 (26.5%)	31 (19.1%)	47 (35.6%)		49 (23.8%)	24 (23.3%)	25 (24.3%)	
Others	14 (4.8%)	10 (6.2%)	4 (3.0%)		6 (2.9%)	2 (1.9%)	4 (3.9%)	
Stage (AJCC 8th)				0.381				0.849
IIB	12 (4.1%)	5 (3.1%)	7 (5.3%)		9 (4.4%)	5 (4.9%)	4 (3.9%)	
IIIA	100 (34.0%)	52 (32.1%)	48 (36.4%)		72 (35.0%)	33 (32.0%)	39 (37.9%)	
IIIB	122 (41.5%)	74 (45.7%)	48 (36.4%)		76 (36.9%)	40 (38.8%)	36 (35.0%)	
IIIC	60 (20.4%)	31 (19.1%)	29 (22.0%)		49 (23.8%)	25 (24.3%)	24 (23.3%)	
Radiation dose (Gy)				0.070				0.721
<60	14 (4.8%)	11 (6.8%)	3 (2.3%)		8 (3.9%)	5 (4.9%)	3 (2.9%)	
≥60	280 (95.2%)	151 (93.2%)	129 (97.7%)		198 (96.1%)	98 (95.1%)	100 (97.1%)	

CRT, Chemoradiotherapy; ECOG PS, Eastern Cooperative Oncology Group performance status.

**Table 2:** Patients characteristics before PSM.

Characteristics	Overall	I-CRT-I group	CRT-I group
	N=294, (%)	N=162, (%)	N=132, (%)
T stage			
T1	41 (13.9%)	18 (11.1%)	23 (17.4%)
T2	90 (30.6%)	50 (30.9%)	40 (30.3%)
T3	47 (16.0%)	23 (14.2%)	24 (18.2%)
T4	116 (39.5%)	71 (43.8%)	45 (34.1%)
N stage			
N0	8 (2.7%)	4 (2.5%)	4 (3.0%)
N1	28 (9.5%)	14 (8.6%)	14 (10.6%)
N2	156 (53.1%)	88 (54.3%)	68 (51.5%)
N3	102 (34.7%)	56 (34.6%)	46 (34.8%)
Induction immunotherapy			
Yes	162 (100%)	162 (100%)	0
No	132 (100%)	0	132 (100%)
Induction chemotherapy			
Yes	253 (86.1%)	157 (96.9%)	96 (72.7%)
No	41 (13.9%)	5 (3.1%)	36 (27.3%)
Concurrent CRT			
Concurent CRT	231 (78.6%)	120 (74.1%)	111 (84.1%)
Sequential CRT	55 (18.7%)	39 (24.1%)	16 (12.1%)
Only-RT	8 (2.7%)	3 (1.9%)	5 (3.8%)
Induction ICIs			
PD-L1 ICIs	9 (3.1%)	9 (5.6%)	
PD-1 ICIs	153 (52.0%)	153 (94.4%)	
Consolidation ICIs			
PD-L1 ICIs	82 (27.9%)	16 (9.9%)	66 (50.0%)
PD-1 ICIs	212 (72.1%)	146 (90.1%)	66 (50.0%)
Best response to induction immunotherapy			
CR	1 (0.3%)	1 (0.6%)	
PR	114 (38.8%)	114 (70.4%)	
SD	43 (14.6%)	43 (26.5%)	
PD	4 (1.4%)	4 (2.5%)	
Best response to CRT			
CR	7 (2.4%)	5 (3.1%)	2 (1.5%)
PR	246 (83.7%)	135 (83.3%)	111 (84.1%)
SD	41 (13.9%)	22 (13.6%)	19 (14.4%)
PD-L1 expression			
<1%	31 (10.5%)	19 (11.7%)	12 (9.1%)
1-49%	41 (13.9%)	19 (11.7%)	22 (16.7%)
≥50%	34 (11.6%)	19 (11.7%)	15 (11.4%)
Missing	188 (63.9%)	105 (64.8%)	83 (62.9%)
The interval days*			
1–42 days	159 (54.1%)	98 (60.5%)	61 (46.2%)
>42 day	135 (45.9%)	64 (39.5%)	71 (53.8%)

CRT, chemoradiation; ICIs, immune checkpoint inhibitors; ECOG, Eastern Cooperative Oncology Group; CR, complete response; PR, partial response; SD,stable disease; PD, progressive disease; PD-L1, programmed death-ligand 1; PD-1, programmed death-1;

* The interval days were the days from CRT completion to the first course of immunotherapy.

Before PSM, the median patient age was 63 years (range, 46–81) and 64 years (range, 33–84) in the I-CRT-I and CRT-I groups, respectively. Higher percentages of squamous cell carcinoma (74.7%) were observed in the I-CRT-I group than in the CRT-I group (61.4%, *p*=0.004). The I-CRT-I group had more patients with stage IIIB disease (45.7% *vs.* 36.4%) and those who received a radiation dose <60 Gy (6.8% *vs.* 2.3%). A total of 103 patients (63.6%) in the I-CRT-I group had bulky tumors, defined as a primary tumor ≥ 5 cm in the greatest dimension or regional lymph nodes ≥ 2 cm in the shortest diameter (18–20).

After PSM, patient characteristics, including sex, age, ECOG PS, smoking history, histology, stage, and radiation dose, were well balanced between the two groups (**Table 1**).

### 3.2 Treatment

All patients in the I-CRT-I group received induction and consolidation immunotherapy, whereas 157 patients (96.9%) received immunochemotherapy. A median of three induction immunotherapy cycles (range 1–8) was administered. Induction ICIs targeted PD-L1 (5.6%, n=9) and PD-1 (94.4%, n=153). Similarly, consolidation ICIs targeted PD-L1 (9.9%, n=16) and PD-1 (90.1%, n=146) in the I-CRT-I group. In the CRT-I group, all patients received consolidation immunotherapy targeting PD-L1 (50.0%, n=66) and PD-1 (50.0%, n=66).

By the time of analysis, 42.6% (69/162) of patients in the I-CRT-I group had received ≥1 year of consolidation immunotherapy. Conversely, 57.4% (93/162) discontinued consolidation immunotherapy due to disease progression (n=31, 19.1%), adverse events (AEs; n=45, 27.8%), and others (n=17, 10.5%). The most common AE leading to discontinuation was pneumonitis (n=31, 19.1%), including radiation pneumonitis and immune-related pneumonitis. In the CRT-I group, 57 patients (43.2%) received consolidation immunotherapy beyond 1 year, whereas 75 patients (56.8%) discontinued it. The reasons for discontinuation included disease progression (n=34, 25.8%), AEs (n=32, 24.2%), and others (n=9, 6.8%). Similarly, the most common AE leading to treatment discontinuation was pneumonitis (n=22, 16.7%).

Within the I-CRT-I group, 157 patients (96.9%) received induction chemotherapy, while definitive concurrent CRT (cCRT), sequential CRT (sCRT), and RT monotherapy were administered to 120 (74.1%), 39 (24.1%), and 3 (1.9%) patients, respectively. In the CRT-I group, induction chemotherapy was administered to 96 patients (72.7%), with definitive cCRT, sCRT, and RT monotherapy utilized in 111 (84.1%), 16 (12.1%), and 5 (3.8%) cases, respectively.

### 3.3 Tumor response

Patients receiving induction immunochemotherapy in the I-CRT-I group exhibited an ORR of 71.0% (n=115) and a DCR of 97.5% (n=158). Specifically, 60, 31, and 50 patients underwent two, three, and four or more cycles of induction immunochemotherapy, respectively. No significant disparities were observed in either the ORR or DCR when comparing two cycles with three or more cycles (ORR: 76.7% vs. 80.6%, *p*=0.664; DCR: 98.3% vs. 96.8%, *p*=1.000), and likewise, when comparing two cycles with four or more cycles (ORR: 76.7% vs. 90.0%, *p*=0.065; DCR: 98.3% vs. 96.0%, *p*=0.873).

### 3.4 Survival outcomes before PSM

**Figure 2:**
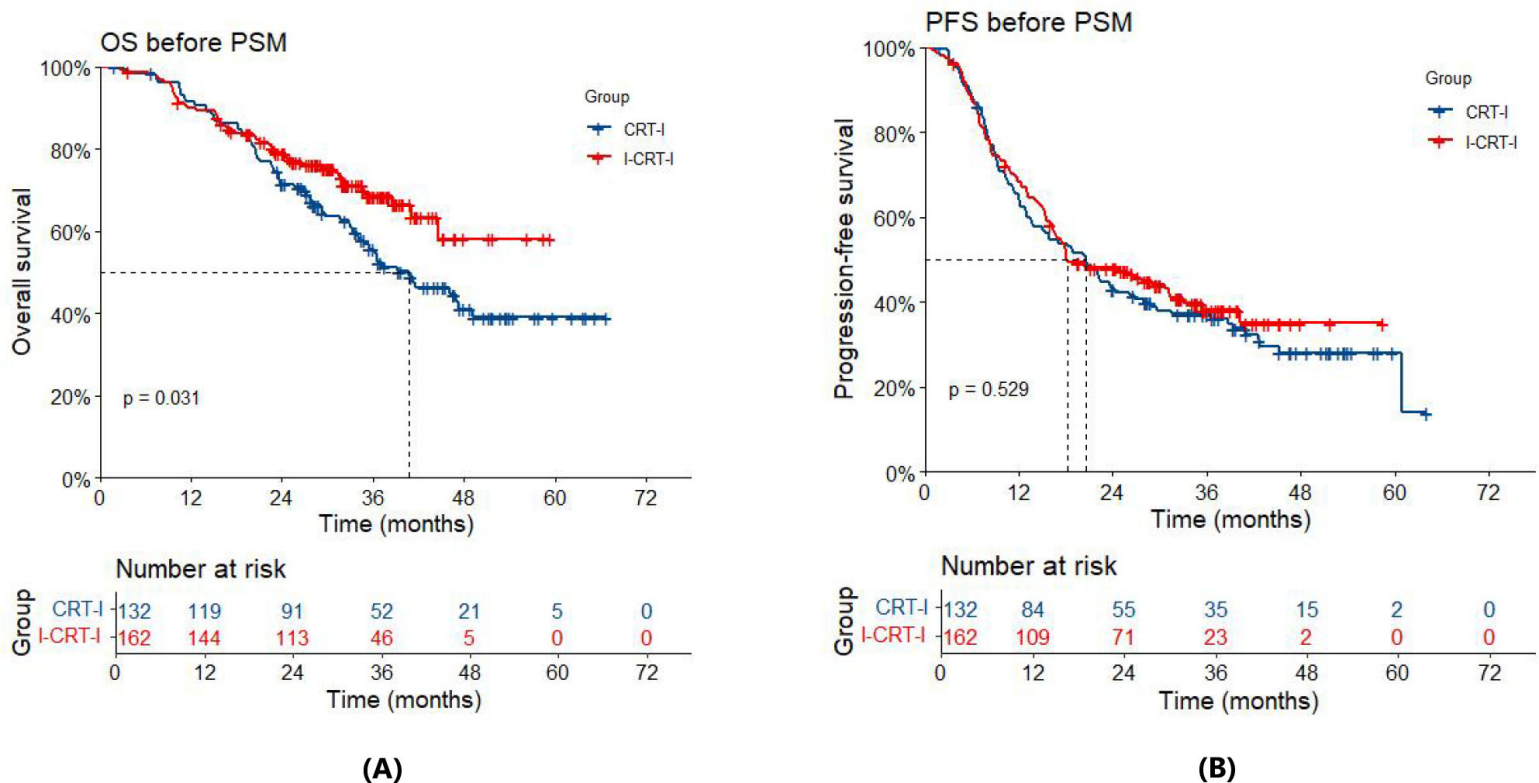
**(A)** Overall survival (OS) of the I-CRT-I group and the CRT-I group before PSM. **(B)** Progression-free survival (PFS) of the I-CRT-I group and the CRT-I group before PSM.

Prior to PSM, the overall cohort had a median follow-up of 37.0 months (34.6 months in the I-CRT-I group and 45.2 months in the CRT-I group). By the time of the analysis, 47 deaths (29.0%) had occurred in the I-CRT-I group and 66 (50.0%) in the CRT-I group. The I-CRT-I group demonstrated a significant improvement in OS compared with the CRT-I group (hazard ratio [HR]=0.66, 95% confidence interval [CI], 0.45–0.96; *p*=0.032). Survival metrics revealed a median OS of not reached (NR) (95% CI: 44.7–Not Available [NA]) in the I-CRT-I group, with corresponding 1-, 2-, and 3-year OS rates of 90.1% (95% CI: 85.6%–94.8%), 79.0% (95% CI: 72.9%–85.7%), and 68.6% (95% CI: 61.0%–77.2%), respectively, while the CRT-I group showed a shorter median OS of 40.8 months (95% CI: 34.1–NA), accompanied by lower survival rates of 91.5% (95% CI: 86.9%–96.5%), 71.5% (95% CI: 64.2%–79.7%), and 55.7% (95% CI: 47.4%–65.5%), respectively (**Figure 2A**). Disease progression was documented in 94/162 patients (58.0%) in the I-CRT-I group, compared to 89/132 patients (67.4%) in the CRT-I group. Although the I-CRT-I group showed a trend toward reduced progression risk (HR = 0.91, 95% CI 0.68–1.22; *p*=0.530), no statistically significant improvement in PFS was observed compared with the CRT-I group. The I-CRT-I group demonstrated a median PFS of 18.2 months (95% CI: 16.3–31.3), with 1-, 2-, and 3-year PFS rates of 68.3% (95% CI: 61.5%–75.9%), 48.1% (95% CI: 40.9%–56.5%), and 38.2% (95% CI: 30.7%–47.6%), respectively, while the CRT-I group exhibited a shorter median PFS of 20.6 months (95% CI: 13.8–28.0) and corresponding rates of 64.7% (95% CI: 57.0%–73.4%), 43.1% (95% CI: 35.4%–52.5%), and 37.2% (95% CI: 29.7%–46.6%), respectively (**Figure 2B**).

### 3.5 Survival outcomes after PSM

**Figure 3:**
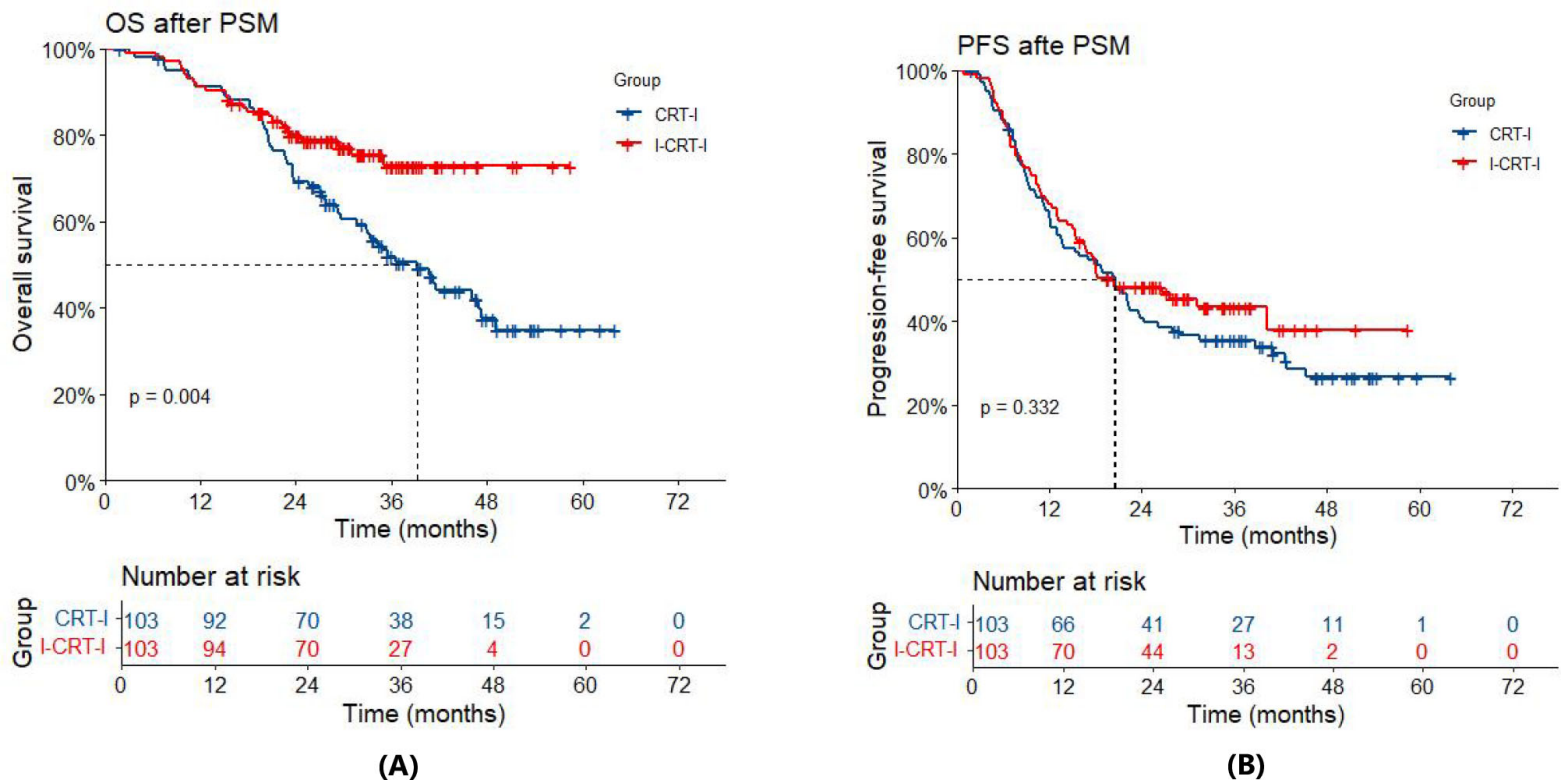
**(A)** Overall survival (OS) of the I-CRT-I group and the CRT-I group after PSM. **(B)** Progression-free survival (PFS) of the I-CRT-I group and the CRT-I group after PSM.

After adjustment using PSM, the entire cohort had a median follow-up of 36.7 months (32.3 months for the I-CRT-I group vs. 44.6 months for the CRT-I group). By the time of the analysis, 24 patients (23.3%) had died in the I-CRT-I group, and 55 patients (53.4%) had died in the CRT-I group. Notably, the I-CRT-I group showed a significant improvement in OS relative to the CRT-I group (HR=0.50, 95% CI 0.31–0.81; *p*=0.005). Survival metrics indicated that the median OS in the I-CRT-I group was NR (95% CI: NA–NA), with 1-, 2-, and 3-year OS rates of 91.3% (95% CI: 86.0%–96.9%), 80.0% (95% CI: 72.5%–88.2%), and 72.9% (95% CI: 63.5%–83.6%), respectively, while the CRT-I group had a shorter median OS of 39.3 months (95% CI: 32.9–49.2), accompanied by lower survival rates of 91.1% (95% CI: 85.7%–96.8%), 69.3% (95% CI: 60.9%–78.9%), and 52.0% (95% CI: 42.8%–63.3%), respectively (**Figure 3A**). Disease progression was observed in 57 out of 103 patients (55.3%) in the I-CRT-I group, as opposed to 70 out of 103 patients (68.0%) in the CRT-I group. Although the I-CRT-I group exhibited a trend toward a reduced progression risk (HR=0.84, 95% CI 0.59–1.20; *p*=0.334), there was no statistically significant improvement in PFS compared with the CRT-I group. The I-CRT-I group had a median PFS of 20.4 months (95% CI: 16.5–NA), with 1-, 2-, and 3-year PFS rates of 68.0% (95% CI: 59.5%–77.6%), 48.3% (95% CI: 39.5%–59.0%), and 43.5% (95% CI: 34.4%–55.0%), respectively, while the CRT-I group presented a median PFS of 20.6 months (95% CI: 13.7–26.1) and corresponding rates of 65.4% (95% CI: 56.8%–75.4%), 40.7% (95% CI: 32.1%–51.5%), and 35.5% (95% CI: 27.3%–46.3%), respectively (**Figure 3B**).

**Supplementary Figure S1** illustrates the subgroup analysis conducted after PSM. In terms of OS, the I-CRT-I group exhibited a significantly improvement compared with the CRT-I group for stage T4 (HR=0.34, 95% CI 0.15–0.78, *p*=0.011), stage N3 (HR=0.36, 95% CI 0.15–0.86, *p*=0.021), and stage IIIB (HR=0.43, 95% CI 0.21–0.90, *p*=0.025). The I-CRT-I group showed an improvement trend compared with the CRT-I group for stage IIIC (HR=0.40, 95% CI 0.141–1.14, *p*=0.086) (**Supplementary Figure S1A**). Regarding PFS, there were no significant differences between the two groups for stage T, stage N or stage (**Supplementary Figure S1B**).

### 3.6 Recurrence pattern

**Figure 4:**
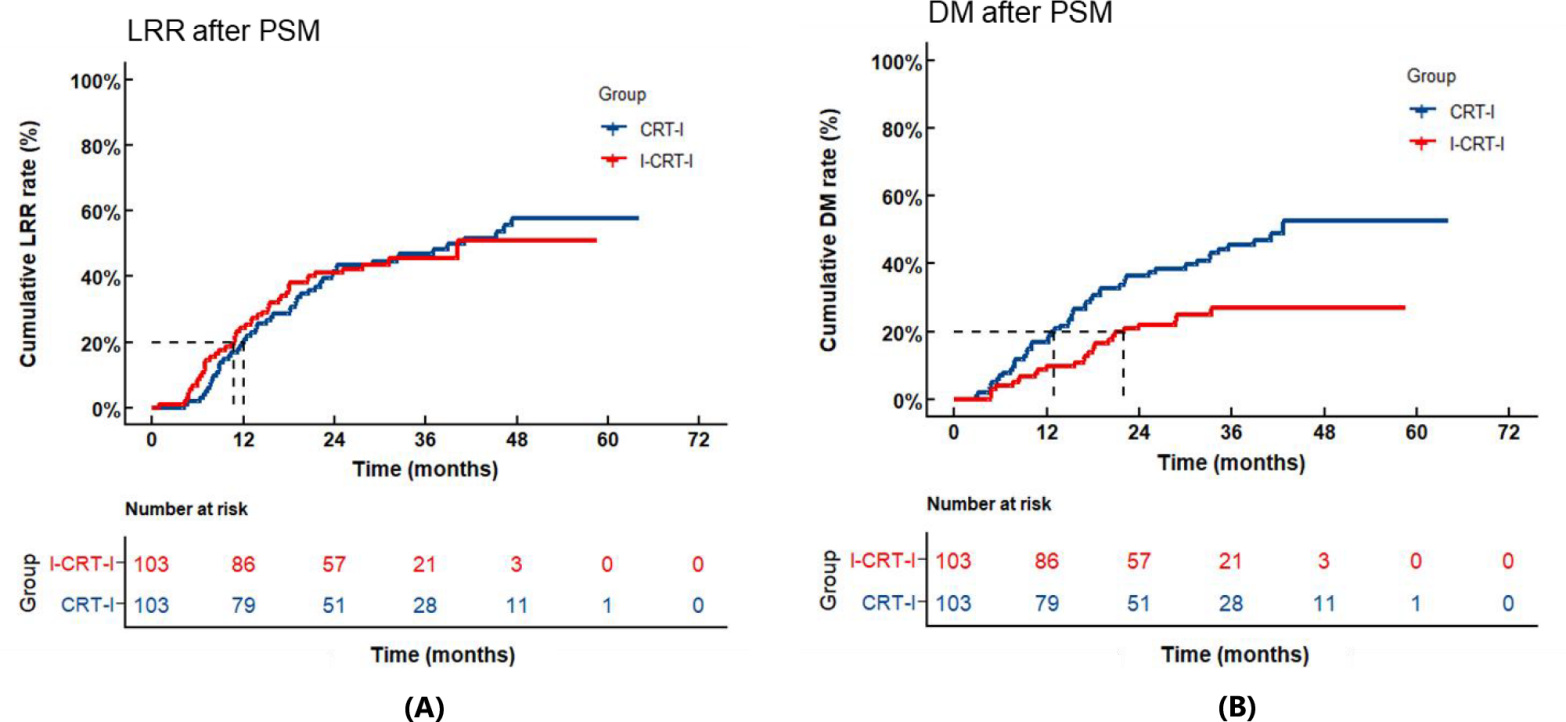
**(A)** Cumulative locoregional recurrence (LRR) of the I-CRT-I group and the CRT-I group after PSM. **(B)** Cumulative distant metastasis (DM) of the I-CRT-I group and the CRT-I group after PSM.

Following PSM adjustment, the I-CRT-I group exhibited 52 recurrence cases (50.5% of the cohort), with 46 patients (44.7%) demonstrating LRR and 25 patients (24.3%) developing DM, comprising LRR-only (n=27, 26.2%), DM-only (n=6, 5.8%), and concurrent LRR plus DM failures (n=19, 18.4%). In contrast, the CRT-I group showed higher recurrence rates (n=66, 64.1%), with 53 patients (51.3%) experiencing LRR and 49 patients (47.6%) presenting DM (**Table 3**), categorized as LRR-only (n=17, 16.5%), DM-only (n=13, 12.6%), and concurrent LRR plus DM failures (n=36, 35.0%). No significant difference was observed in cumulative LRR (*p*=0.940) between the two groups. The 1-, 2-, and 3-year cumulative LRR rates were 24.3% (95% CI: 16.5%–32.9%), 41.0% (95% CI: 31.4%–50.4%), and 45.6% (95% CI: 35.1%–55.5%) in the I-CRT-I group, and 19.8% (95% CI: 12.6%–28.1%), 41.6% (95% CI: 31.8%–51.0%), and 47.0% (95% CI: 36.8%–56.5%) in the CRT-I group, respectively (**Figure 4A**). Significantly lower cumulative DM (*p*=0.004) was noted for the I-CRT-I group compared with the CRT-I group. Specifically, the 1-, 2-, and 3-year cumulative DM rates were 9.7% (95% CI: 4.9%–16.4%), 22.0% (95% CI: 14.4%–30.6%), and 27.1% (95% CI: 18.1%–37.0%), respectively, in the I-CRT-I group and 16.8% (95% CI: 10.2%–24.7%), 36.6% (95% CI: 27.2%–46.0%), and 45.5% (95% CI: 35.3%–55.2%), respectively, in the CRT-I group (**Figure 4B**).

**Table 3:** Recurrence patterns in two groups before PSM.

Recurrence patterns	Beore PSM		After PSM	
	I-CRT-I	CRT-I	I-CRT-I	CRT-I
	N = 162, (%)	N = 132, (%)	N = 103, (%)	N = 103, (%)
Total recurrence	78 (48.1%)	81 (61.4%)	52 (50.5%)	66 (64.1%)
LRR	75 (46.3%)	62 (47.0%)	46 (44.7%)	53 (51.5%)
DM	42 (25.9%)	62 (47.0%)	25 (24.3%)	49 (47.6%)

### 3.7 Safety

Before adjustment using PSM, treatment-related AEs (TRAEs) of any cause and grade occurred in 96.3% and 95.5% of patients in the I-CRT-I and CRT-I group, respectively (**Table 4**). Most of these events were mild to moderate, with grade1–2 AEs occurring in 152 patients (93.8%) in the I-CRT-I group and 124 patients (94.0%) in the CRT-I group. The I-CRT-I group exhibited a significantly higher incidence of anemia that the CRT-I group (53.7% vs. 26.5%; *p*<0.001), while other AEs showed no statistically significant differences between the two groups. In the I-CRT-I group, 93 out of 162 patients (57.4%) experienced any grade of pneumonitis (radiation pneumonitis and immune-related pneumonitis), including grade 1 (n=34, 21.0%), grade 2 (n=43, 26.5%), grade 3–4 (n=14, 8.6%), and grade 5 (n=2, 1.2%). Thirty-one patients (19.1%) discontinued consolidation immunotherapy due to the occurrence of grade 2 or higher pneumonitis. Within the CRT-I group, 77 out of 132 patients (58.3%) developed any grade of pneumonitis, including grade 1 (n=48, 36.4%), grade 2 (n=23, 17.4%), and grade 3–4 (n=6, 4.5%). Twenty-two patients (16.7%) discontinued consolidation immunotherapy due to the occurrence of grade 2 or higher pneumonitis. The I-CRT-I group experienced a higher incidence of grade 3–5 pneumonitis than the CRT-I group (9.9% vs. 4.5%, *p*=0.084). In the entire cohort (n=294), patients receiving consolidation immunotherapy of PD-1-targeting ICIs (n=212, 72.1%) had a higher incidence of any-grade or grade 3–5 pneumonitis than those who received PD-L1-targeting ICIs (n=82, 27.9%), including any-grade pneumonitis (58.5% vs. 56.1%, *p*=0.913) and grade 3–5 pneumonitis (8.5% vs. 4.9%, *p*=0.291).

**Table 4:** Adverse events between the two treatments groups before PSM.

Toxicities	I-CRT-I group				CRT-I group				*P*
	N=162, (%)				N=132, (%)				
	All Grade	Grade 1-2	Grade 3-4	Grade 5	All Grade	Grade 1-2	Grade 3-4	Grade 5	
Any event	156 (96.3%)	152 (93.8%)	57 (35.2%)	2 (1.2%)	126 (95.5%)	124 (94.0%)	48 (36.4%)	1 (0.8%)	
Anemia	87 (53.7%)	84 (51.9%)	3 (1.9%)	0	35 (26.5%)	32 (24.2%)	3 (2.3%)	0	**<0.001**
Leukopenia	114 (70.4%)	90 (55.6%)	24 (14.8%)	0	97 (73.5%)	70 (53.0%)	27 (20.5%)	0	0.555
Neutropenia	82 (50.6%)	60 (37.0%)	22 (13.6%)	0	71 (53.8%)	42 (31.8%)	29 (22.0%)	0	0.588
Thrombocytopenia	53 (32.7%)	40 (24.7%)	13 (8.0%)	0	49 (37.1%)	42 (31.8%)	7 (5.3%)	0	0.43
Esophagitis	114 (70.3%)	89 (54.9%)	25 (15.4%)	0	87 (65.9%)	69 (52.3%)	18 (13.6%)	0	0.747
Pneumonitis	93 (57.4%)	77 (47.5%)	14 (8.6%)	2 (1.2%)	77 (58.3%)	71 (53.8%)	6 (4.5%)	0	0.873
Hypothyroidism(irAEs)	16 (9.9%)	15 (9.3%)	1 (0.6%)	0	23 (17.4%)	22 (16.7%)	1 (0.8%)	0	0.058
Rash(irAEs)	11 (6.8%)	10 (6.2%)	1 (0.6%)	0	5 (3.8%)	5 (3.8%)	0	0	0.259
Hepatitis(irAEs)	11 (6.8%)	9 (5.6%)	2 (1.2%)	0	6 (4.5%)	6 (4.5%)	0	0	0.412
Enteritis(irAEs)	1 (0.6%)	1 (0.6%)	0	0	3 (2.3%)	2 (1.5%)	0	1 (0.8%)	>0.999
Myocarditis(irAEs)	3 (1.9%)	3 (1.9%)	0	0	7 (5.3%)	7 (5.3%)	0	0	0.119
Endocrinopathies(irAEs)	2 (1.2%)	1 (0.6%)	1 (0.6%)	0	3 (2.3%)	2 (1.5%)	1 (0.8%)	0	0.66
Renal toxicity(irAEs)	1 (0.6%)	1 (0.6%)	0	0	2 (1.5%)	2 (1.5%)	0	0	0.589
Peripheral neuropathy(irAEs)	4 (2.5%)	4 (2.5%)	0	0	0	0	0	0	0.13

irAEs, immune-related adverse events;

## 4 Discussion

Our study showed that among patients with unresectable stage III NSCLC, a statistically significant improvement in OS was observed with the integration of induction immunochemotherapy before definitive CRT and consolidation immunotherapy. However, no improvement was observed in PFS. To our knowledge, this multi-institutional retrospective analysis represents the largest real-world cohort to date evaluating the efficacy of induction immunochemotherapy before definitive CRT and consolidation immunotherapy for unresectable stage III NSCLC.

Although consolidation immunotherapy after definitive cCRT yielded encouraging survival benefits, further improvement in its effect is expected. Optimizing the PACIFIC treatment regimen has consistently been a focal point and challenge in the field of unresectable stage III NSCLC, with the integration of induction immunotherapy being a key area of exploration. An undamaged tumor might contain more neoantigens for priming the immune system (12, 13), which could enable effective T-cell infiltration and promote the immune system (21). In the prospective phase II AFT-16 study, 62 patients received 2–4 cycles of induction immunotherapy followed by definitive cCRT and consolidation atezolizumab. Patients who exhibited disease progression after two cycles of induction immunotherapy immediately received definitive cCRT. The results showed a median PFS of 30.0 months and 12- and 24-month PFS of 68.9% and 54.2%, respectively, and a median OS of NR and 12- and 24-month OS of 87.0% and 73.7%, respectively (15). The phase II KEYNOTE-799 study, which administered one cycle of induction pembrolizumab plus chemotherapy followed by definitive cCRT and concurrent and consolidated pembrolizumab, also reported positive survival outcomes. The median PFS in Cohort A was 30.6 months, with 1-year and 2-year PFS rates of 67.3% and 55.3%, respectively. In Cohort B, the median PFS was NR, and the 1-year and 2-year PFS rates were 69.4% and 60.6%, respectively. The median OS in Cohort A was NR, with 1-year and 2-year OS rates of 81.3% and 64.3%, respectively. In Cohort B, the median OS was NR, and the 1-year and 2-year OS rates were 88.2% and 71.2%, respectively (22). At the 2024 World Conference on Lung Cancer, the oral report of the phase II APOLO trial (definitive cCRT combined with induction and consolidation atezolizumab) showed promising survival outcomes (median PFS: 20.8 months; 12-month PFS: 68.4%; 18-month PFS: 60.5%; 12-month OS: 86.8%) (23). In our study, the I-CRT-I cohort demonstrated a median PFS of 20.4 months, with 1-, 2-, and 3-year PFS rates of 68.0%, 48.3% and 43.5%, respectively, alongside 1-, 2- and 3-year OS rates of 91.3%, 80.0% and 72.9%, respectively. Thus, the survival outcomes found in our study were similar to those in the three aforementioned prospective studies, with the addition of induction immunotherapy demonstrating encouraging survival outcomes. Compared with the consolidation immunotherapy arm in the PACIFIC trial (1-, 2- and 3-year OS rate: 83.1%, 66.3% and 56.7%) (6) and in the GEMSTONE-301 trial (1- and 2-year OS rate: 86.0% and 67.6%) (24), the I-CRT-I group in our study demonstrated a relatively high OS rate. The I-CRT-I group showed significantly improved OS in patients with stages T4, N3, IIIB, and IIIC, who had a poor prognosis with higher tumor burden. Compared with the consolidation immunotherapy arm in the PACIFIC trial (3-year PFS rate: 39.7%), the I-CRT-I group in our study demonstrated that the integration of induction immunotherapy did not significantly improve the PFS rate. The results of the above trials and the current study support induction and consolidation immunotherapy as a promising treatment strategy. In the future, large-scale randomized controlled trials (RCTs) will be necessary to further demonstrate the role and efficacy of induction immunotherapy in unresectable stage III NSCLC.

Induction immunochemotherapy can reduce tumor volume and burden (7, 9), thus improving the completion rate of definitive CRT and decreasing the radiation field. Therefore, induction immunochemotherapy before definitive CRT is increasingly recommended for patients who cannot tolerate definitive cCRT because of their bulky tumor volume, high tumor burden, or a strong desire for surgery in the real world. In our study, bulky tumors constituted 63.6% of patients in the I-CRT-I group. The ORR and DCR for induction immunochemotherapy were 71.0% and 97.5%, respectively. These rates did not differ significantly between patients receiving 2 vs. ≥3 cycles or ≥4 cycles of induction immunochemotherapy. The reduction in tumor volume after induced immunochemotherapy enables patients to tolerate definitive cCRT. Moreover, receiving ≥3 cycles of induction immunochemotherapy may not significantly increase the tumor response rate but may cause more immunochemotherapy-related AEs. Wang et al. also proposed that two cycles of induction immunochemotherapy should be considered for bulky stage III NSCLC to maximize tumor downsizing (18). The optimal number of induction treatment cycles remains unclear; therefore, future high-level trials are needed to answer this question.

In our study, the I-CRT-I group exhibited a significantly lower cumulative DM than the CRT-I group after PSM. A preclinical study revealed that activation of the immune system yielded more effective immune surveillance against micrometastatic disease (14). A retrospective study in China also showed induction plus consolidation ICIs might reduce DM (25), no significant difference was observed in cumulative LRR between the two groups. Locoregional failure emerged as the predominant recurrence pattern in patients with unresectable stage III NSCLC undergoing a sequential treatment protocol of induction immunochemotherapy followed by definitive CRT and subsequent consolidation immunotherapy. However, the relatively small sample size due to the retrospective nature of the study limited our ability to draw definitive conclusions. Future prospective RCTs are required to address these issues.

Our study revealed no difference in the incidence of grade ≥3 TRAEs between the I-CRT-I (59 patients, 36.4%) and CRT-I groups (49 patients, 37.1%). The AFT-16 study showed that 30 patients (48.4%) and 17 patients (27.4%) developed grade ≥3 TRAEs and grade ≥3 immune-related AEs (irAEs), respectively (15). Therefore, the combination of induction immunotherapy and definitive CRT followed by consolidation immunotherapy demonstrated tolerable toxicity. Compared with the CRT-I group, the I-CRT-I group had a higher incidence of anemia (53.7% vs. 26.5%), which is associated with the fact that more patients in the I-CRT-I group received induction chemotherapy (96.9% vs. 72.7%). The incidence of pneumonitis did not differ between the two groups in our study (57.4% vs. 58.3%). However, the incidence of grade 3–5 pneumonitis was higher in the I-CRT-I group than in the CRT-I group (9.9% vs. 4.5%). Thus, induction immunotherapy may be associated with increased rates of grade 3–5 pneumonitis. Induction immunotherapy also resulted in a higher rate of grade 3–5 pneumonitis in the KEYNOTE-799 study (cohort A, 8.0%; cohort B, 6.9%) (16, 22) than in the PACIFIC study (3.4%) (2). A Chinese retrospective study also reported significantly higher rates of grade 3–5 pneumonitis in patients receiving induction plus consolidation immunotherapy than in those receiving consolidation immunotherapy (14.7% vs. 3.7%, *p*=0.039) (25). The higher rate of grade 3–5 pneumonitis in the I-CRT-I group in this study may be attributed to the increased use of PD-1 inhibitors during induction and consolidation immunotherapy, with a significantly greater proportion of patients receiving PD-1 inhibitors in this group compared with the CRT-I group (90.1% vs. 50.0%) during consolidation immunotherapy. Preclinical and clinical studies consistently demonstrated that PD-1 inhibitors are more likely to induce pneumonitis than PD-L1 inhibitors (26, 27). The incidence of any-grade pneumonitis (57.4%, 58.3%) and grade 3–5 pneumonitis (9.9%, 4.5%) in both groups in our study was higher than that in the PACIFIC study (33.9%, 3.4%) (2). The reduced incidence of pulmonary toxicity observed in the PACIFIC trial may be attributed to differences in patient characteristics, given that the study excluded patients with pre-existing grade ≥2 pneumonitis from prior CRT (2), thereby removing a high-risk subpopulation predisposed to developing pulmonary complications. This difference may also be attributed to racial factors. In a real-world Korean study, the incidence of any-grade and grade 3 pneumonitis was 81.0% and 14.3%, respectively, among Asian patients receiving durvalumab consolidation therapy (28). Conversely, a multi-ethnic Canadian real-world study reported lower rates of any-grade (29.9%) and grade 3–5 (6.1%) pneumonitis associated with durvalumab consolidation (29). Furthermore, a real-world meta-analysis observed a significantly lower incidence of any-grade pneumonitis in Western studies than in Asian studies (22% vs. 62%; *p*=0.017) (30). All patients included in the present study were Asian, whereas these comprised only 25.2% of patients receiving durvalumab consolidation in the PACIFIC study (2). These ethnic differences may contribute to variations in tolerance to pulmonary toxicity and sensitivity to ICI therapy due to genetic disparities among individuals. For instance, Asian patients exhibit higher rates of EGFR-sensitizing mutations, ranging from 40% to 50%, compared with 10% to 15% in non-Asian patients (31). This discrepancy could also be attributed to the use of different checkpoint inhibitors. In our study, PD-1 inhibitors were administered in addition to consolidation immunotherapy in 72.1% of the patients and PD-L1 inhibitors in 27.9%; however, in the PACIFIC trial, all patients in the durvalumab group received PD-L1 inhibitors. As previously mentioned, PD-1 inhibitors are more likely to cause pneumonitis than PD-L1 inhibitors. Thus, the significantly higher incidence of pneumonitis observed in our study may be related to the use of PD-1 inhibitors. Additionally, the overlapping period between this study (2018–2021) and the COVID-19 pandemic may have led to the identification of more pneumonitis cases (32).

Our study has several limitations. First, this was a retrospective study. Although we used PSM to reduce bias between the two groups, future prospective RCTs are needed to verify our findings. Second, delayed response evaluations and cancer follow-up examinations are common in retrospective studies, leading to PFS overestimation and toxicity underestimation. Third, the patients in this study were treated with multiple ICIs rather than a single ICIs, potentially affecting prognosis and toxicity. Fourth, this study did not exclude patients with non-sensitive mutations of EGFR mutations, which might have some impact on survival outcomes. Additionally, because PD-L1 immunohistochemical expression was not routinely measured in patients with stage III NSCLC at our center before December 2022, analyses of PD-L1 expression and efficacy were not applicable.

## 5 Conclusion

Our study demonstrated that for unresectable stage III NSCLC, induction immunochemotherapy followed by definitive CRT and consolidation immunotherapy may improve OS and decrease DM, compared with the conventional approach of definitive CRT followed by consolidation immunotherapy, while maintaining a manageable safety profile. Therefore, further prospective trials must be conducted to rigorously validate the role and value of induction immunochemotherapy, providing more conclusive evidence regarding its efficacy and safety in treating this disease.

## Data Availability

The original contributions presented in the study are included in the article/**Supplementary Material**. Further inquiries can be directed to the corresponding authors.
