# Supplementary material for: Induction immunochemotherapy followed by definitive chemoradiotherapy and consolidation immunotherapy for unresectable stage III non-small cell lung cancer: a multi-institutional retrospective cohort study
Source: Front Immunol. 2025 Jul 31;16:1602082. doi: 10.3389/fimmu.2025.1602082 (PMC12350322; doi:10.3389/fimmu.2025.1602082)
Supplement: Supplementary file 1 [file DataSheet1.docx]

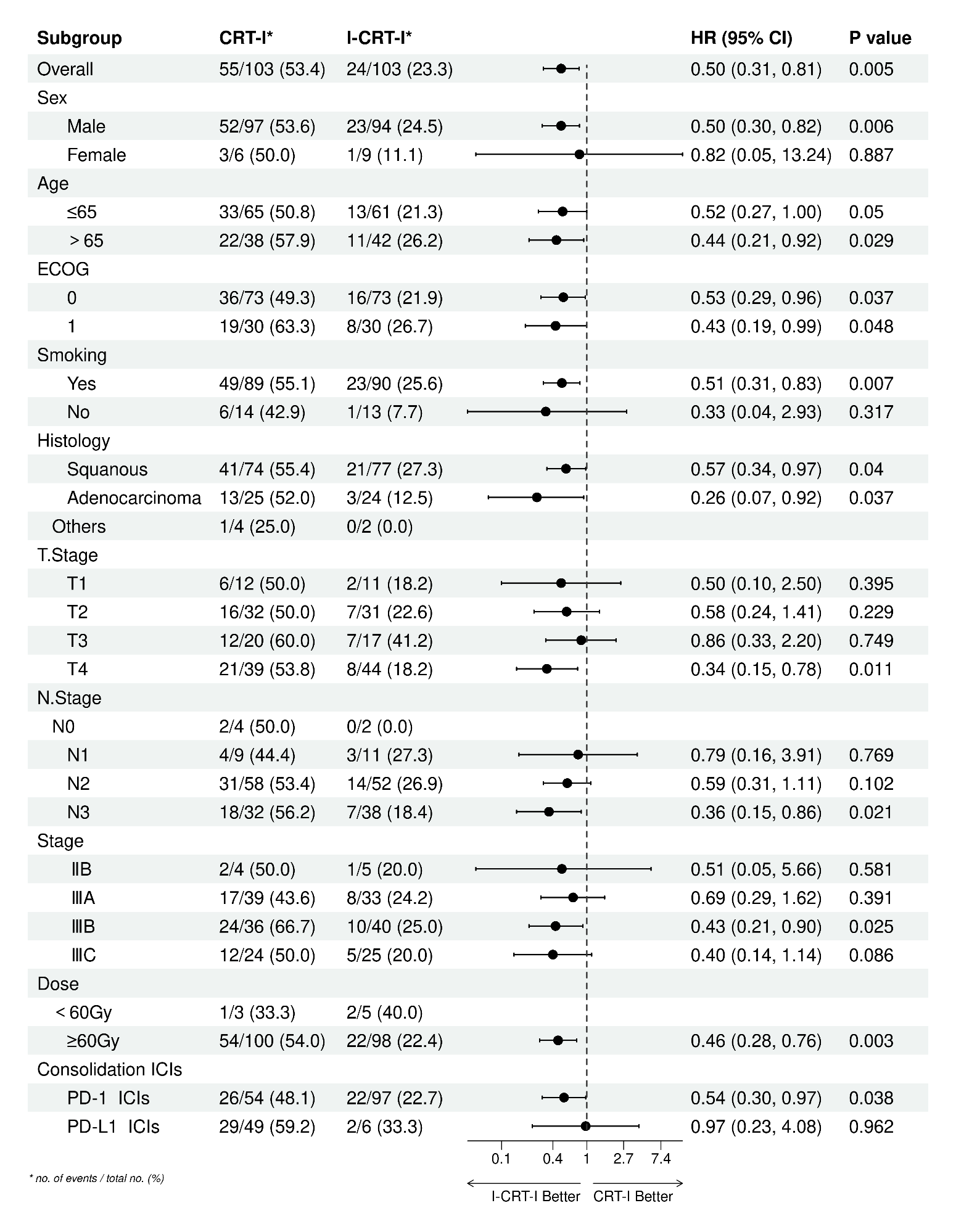


**Figure S1A. Hazard ratios for subgroups of overall survival (OS)** **comparing the I-CRT-I group to the CRT-I group after PSM.**


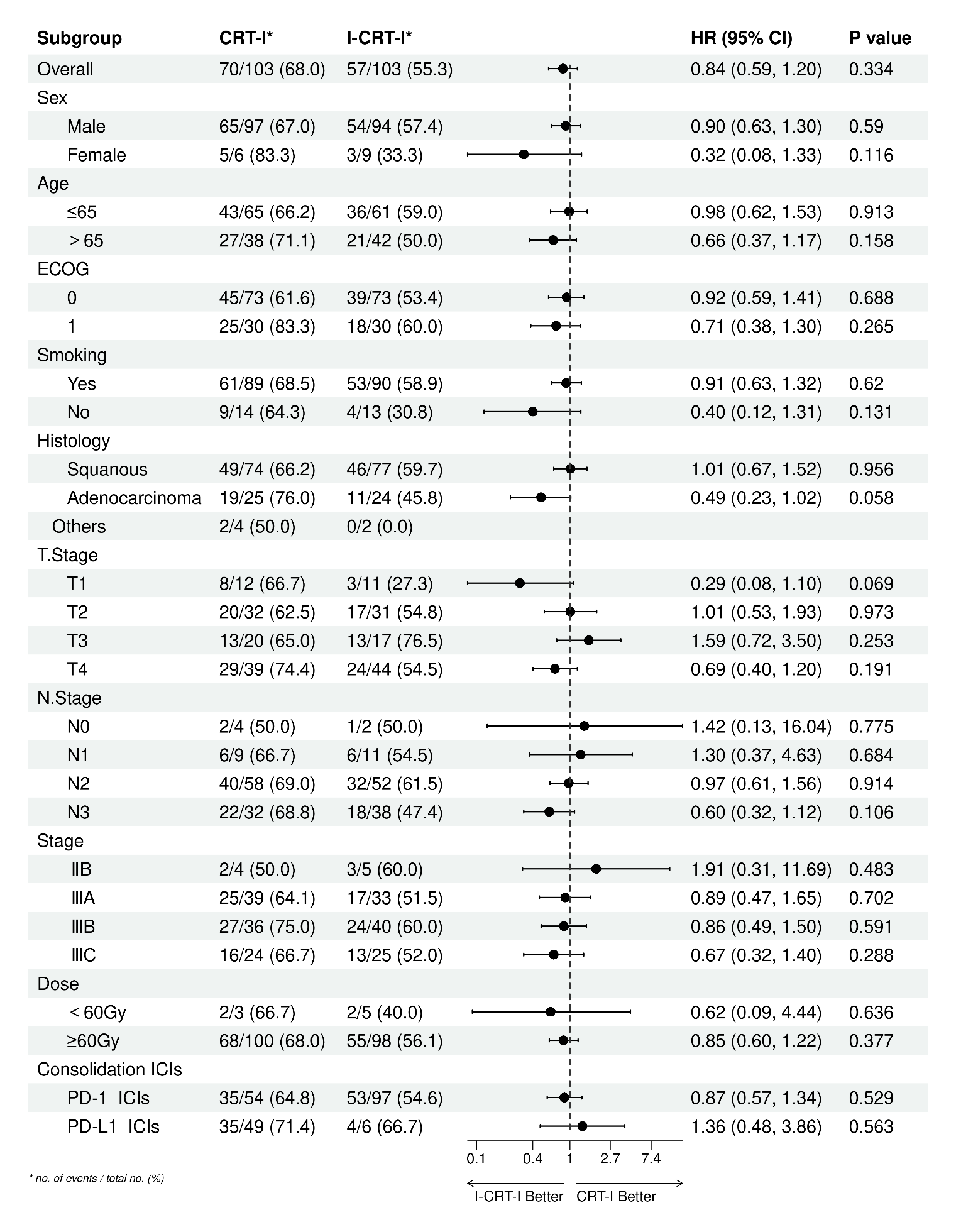


**Figure S1B. Hazard ratios for subgroups of progression-free survival (PFS) comparing the I-CRT-I group to the CRT-I group after PSM.**
